# *Borrelia miyamotoi*–Associated Neuroborreliosis in Immunocompromised Person

**DOI:** 10.3201/eid2209.152034

**Published:** 2016-09

**Authors:** Katharina Boden, Sabine Lobenstein, Beate Hermann, Gabriele Margos, Volker Fingerle

**Affiliations:** University Hospital Jena, Jena, Germany (K. Boden, B. Hermann);; Burgenlandkreis Hospital Naumburg, Naumburg, Germany (S. Lobenstein);; Bavarian Health and Food Safety Authority, Oberschleissheim, Germany (G. Margos, V. Fingerie)

**Keywords:** vector-borne infections, neuroborreliosis, Borrelia miyamotoi, immunocompromised persons, ticks, bacteria, Germany, Lyme neuroborreliosis

## Abstract

*Borrelia miyamotoi* is a newly recognized human pathogen in the relapsing fever group of spirochetes. We investigated a case of *B. miyamotoi* infection of the central nervous system resembling *B. burgdorferi*–induced Lyme neuroborreliosis and determined that this emergent agent of central nervous system infection can be diagnosed with existing methods.

The tickborne relapsing fever spirochete *Borrelia miyamotoi* was described in 1994 but only recently was recognized as a human pathogen (*1*). This spirochete is present in *Ixodes* ticks across the Northern Hemisphere, comparable to the distribution of *B. burgdorferi* sensu lato, but reported cases are still rare (*2**,**3*). Thus far, only 2 cases of central nervous system infection have been reported, both with a chronic course and both in immunocompromised persons (*4**,**5*). We investigated a case of *B. miyamotoi* infection that was initially diagnosed as seronegative early Lyme neuroborreliosis (LNB) that could be attributed to *B. miyamotoi* only by molecular–biological methods.

## The Case

On July 31, 2015, a 74-year-old woman with a history of non-Hodgkin lymphoma (follicular type, stage IV) came to the Burgenlandkreis Hospital (Naumburg, Germany). She reported dizziness, vomiting, and a headache of 5 days’ duration. She had been treated for her lymphoma with cyclophosphamide, doxorubicin, vincristine, and prednisone with rituximab during May–August 2012. Thereafter, she received rituximab maintenance therapy (375 mg/m^2^ every 8 weeks) until November 2014. The monoclonal antibody rituximab that targets the CD20 antigen expressed by B cells leads to anti-CD20–mediated B cell depletion. This treatment, together with the underlying hematologic disease, resulted in an immunocompromised condition. The patient reported 2 tick bites while in Elsteraue municipality (Saxony-Anhalt, Germany) during June and July 2015 but had no recent travel history. On examination, she had slight neck stiffness but no other notable findings.

A lumbar puncture was performed to assess the patient for viral or lymphomatous meningitis. Cerebrospinal fluid (CSF) showed a pleocytosis of 70 leukocytes/μL (reference 0–5 leukocytes/μL) and elevated protein at 1,718 mg/L (reference 150–400 mg/L); albumin quotient (Qalb) 34.8% (reference <9%), lactate 5.58 mmol/L (1.2–2.1 mmo/L), and glucose quotient 0.45 (>0.5); and an intrathecal IgM synthesis of 18%. The greatly elevated lactate and highly increased Qalb and protein made a viral cause unlikely. Pappenheim staining of a cytospin preparation of CSF revealed a mixed cell population (32% polymorphonuclear leukocytes, 61% lymphocytes, and 7% monocytes) with heterogeneous morphology of lymphocytes that did not suggest lymphomatous meningitis. Serum and CSF were negative for *B. burgdorferi*–specific antibodies by the chemiluminescent immunoassay LIASON (DiaSorin, Vercelli, Italy). However, because the CSF constellation was typical for LNB, including the slight intrathecal IgM synthesis (*6*), the patient was treated with 2 intravenous ceftriaxone (2,000 mg 1×/d for 3 wks).

To exclude early tuberculous meningitis, we examined the CSF for mycobacteria by PCR, culture, and staining. To further substantiate the LNB diagnosis, we determined the B-cell attractant chemokine CXCL13 from CSF. The measured value of 1,155 pg/mL was clearly higher than the minimum concentration of 250 pg/mL, indicating neuroborreliosis and thus supporting our working diagnosis of LNB (*7*). After 5 days of treatment, the patient recovered fully. A second lumbar puncture after 7 days of treatment revealed a stable count of 76 monomorphic lymphocytes/μL. In addition, protein, Qalb, and CXCL13 had decreased (Table). However, we remained unable to detect *B. burgdorferi*–specific antibodies in serum or CSF, and *B. burgdorferi*–specific PCR of the first CSF specimen showed no amplification product.

**Table:** CSF findings of *Borrelia miyamotoi* meningitis cases and patients with Lyme neuroborrelioses*

Finding (reference)	Case (reference)			
	New Jersey, USA (*4*)	Netherlands (*5*)	Germany (this study)	Lyme neuroborreliosis(*6*)†
Leukocytes/μL (0–5 cells/μL)	65	388	70	170.5 [57.0–369]
Differential count	23% PMNC, 70% lymphocytes, 6% monocytes, 1% diverse	60% mononuclear cells	32% PMNC, 61% lymphocytes, 7% monocytes	
Protein level, mg/dL (150–400 mg/dL)	>300	486	1718	1,232 [697–1,926]
Qalb, × 10^3^ (<9)			34.8	17.2 [9.7–28.4]
Quantitative IgM, × 10^3^			18.1	Elevated in 70%
Glucose, mmol/L (2.2–4.2 mmol/L)	1.8	1.6	2.41	
Glucose ratio (>0.5)			0.45	
Lactate, mmol/L (1.2–2.1 mmol/L)			5.58	>3.5 in 4%‡
Routine microscopy		Cellular CSF with high nos. of granulocytes and plasma cells	Cellular CSF with heterogeneous morphology	
CXCL13, pg/mL (100 to <250 pg/mL, borderline)			1,150; 8 d after start of therapy: 186	>415 (*7*)
Spirochetes visible in CSF by	Gram staining, Giemsa staining	Darkfield microscopy	Acridinorange staining, not definable at Pappenheim cytospin	Typically negative

*Blank cells indicate value not determined or was within reference range. CSF, cerebrospinal fluid; PMNC, polymorphonuclear leukocytes; Qalb, albumin quotient. †n = 118 patients. Values are median [interquartile range]. ‡Associated with headache.

**Figure 1:**
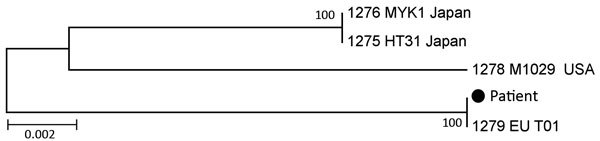
Molecular phylogenetic analysis of *Borrelia* strain from cerebrospinal fluid of a 74-year-old immunocompromised woman in Germany (black dot) conducted by using 6 multilocus sequence typing genes (*clpA*, *clpX*, *pepX*, *pyrG*, *recG*, *rplB*) of *Borrelia miyamotoi*. The sequences obtained from the patient sample clustered with *B. miyamotoi* strain EU_T01 from Europe, retrieved from the PubMLST *Borrelia* database (http://pubmlst.org/borrelia/). The phylogenetic relationship of the sample analyzed was inferred by using DNA sequences of chromosomal housekeeping genes. The maximum-likelihood method based on the general time reversible model (*8*) was applied. The tree with the highest log likelihood (–5531.9051) is shown. The percentage of trees in which the associated taxa clustered is shown next to the branches. Initial tree(s) for the heuristic search were obtained automatically by applying neighbor-joining and BioNJ algorithms (http://bionj.org/about-bionj) to a matrix of pairwise distances estimated by using the maximum composite likelihood approach and then selecting the topology with superior log likelihood value. The tree is drawn to scale; branch lengths are measured in the number of substitutions per site. The analysis involved 5-nt sequences. Codon positions included were 1st+2nd+3rd+Noncoding. All positions containing gaps and missing data were eliminated. The final dataset contained 3,642 positions. Evolutionary analyses were conducted in MEGA5 (*9*).

To conclusively identify the pathogen, we tested the first CSF and serum samples by PCR for panbacterial 16S rRNA, with a positive result from CSF only. Using the BLAST algorithm (http://blast.ncbi.nlm.nih.gov/Blast.cgi) in GenBank, we found, to our surprise, that the sequence was identical to that for *B. miyamotoi*. Moreover, sequences of 16S rRNA, *flaB*, and 6 housekeeping genes revealed the European type of *B. miyamotoi* (Figure 1; details of molecular phylogenetic analysis and GenBank accession numbers in Technical Appendix).

**Figure 2:**
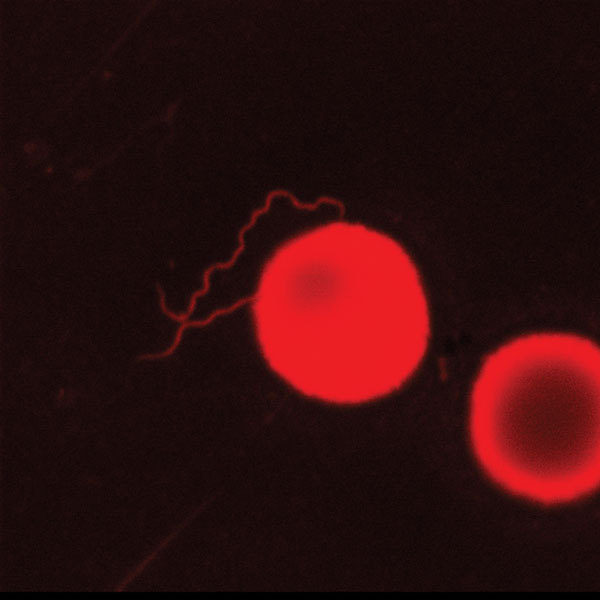
*Borrelia miyamotoi* in cerebrospinal fluid stained by acridine orange (LSM Exciter 5, Zeiss, Germany). The cerebrospinal fluid was from a 74-year-old woman with non-Hodgkin lymphoma. Original magnification ×1,000.

*B. miyamotoi* is one of the *Borrelia* species that causes relapsing fever. The spirochetes of the relapsing fever *Borrelia* group are more easily detectable than *B. burgdorferi* spirochetes from blood and CSF by microscopy and PCR. We therefore reexamined the first Pappenheim-stained cytospin preparation from CSF, but no spirochetes were recognizable. To increase the sensitivity, we restained the preparation with acridine orange. A few spirochetes were then microscopically visible in the preparation using an Axioskop (Zeiss, Germany) (Figure 2).

## Conclusions

The genus *Borrelia* is divided into 2 groups: *B. burgdorferi* sensu lato, which causes Lyme disease, and a group of species that cause relapsing fever. *B. miyamotoi* belongs to the second group and was first described in 2011 as a human pathogen in several patients from Russia who had an unspecific febrile illness where few patients experienced febrile relapses, which is the typical sign for relapsing fever (*1*). *B. miyamotoi* was also reported to have caused meningoencephalitis in a patient from New Jersey, USA (*4*), and a patient from the Netherlands (*5*). As with the patient we report, these patients had a history of non-Hodgkin lymphoma with recent rituximab treatment. Because of the increasing indications for rituximab treatment (*8*) and a prevalence of *B. miyamotoi* in *Ixodes ricinus* ticks in Europe of up to 3.2% (*2*), clinicians should be aware that cases of *B. miyamotoi* neuroborreliosis may increase (*4*).

Detection of specific antibodies is a useful complementary diagnostic method. Only recently it was shown that assays based on the GlpQ protein, which is present in relapsing fever *Borrelia* but absent in *B. burgdorferi* sensu lato, might be useful in diagnosing *B. miyamotoi* infection (*2**,**3*). Moreover, up to 90% of PCR-confirmed *B. miyamotoi* infections were positive in a *B. burgdorferi* enzyme immunoassay, although only a few by 2-tiered testing (reviewed in *3*). For the case we report, we had no access to a GlpQ assay, and the standard serologic tests for Lyme borreliosis were negative, as it was for the 2 published reports (*4**,**5*). However, a clinician relies on combining history, clinical examination, and routine CSF analyses. Clinicians should therefore be aware of possible *B. miyamotoi* neuroborreliosis, especially in patients who have a history of non-Hodgkin lymphoma and recent rituximab treatment. The clinical picture seems to be unspecific. Although the patient we report had acute symptoms (dizziness, vomiting, and headache) of short duration, the other published cases had decline in mental status (slow cognitive processing, memory deficits), and disturbed gait developing gradually over several months. All 3 *B. miyamotoi* cases had CSF pleocytosis with elevated CSF protein concordant with CSF changes found in LNB (Table). We even found slight intrathecal IgM synthesis, as is seen in 70% of patients with LNB (*6*). However, a viral cause must be excluded, especially when CSF protein is only slightly elevated.

In recent years, CXCL13 has been identified as a potentially sensitive and specific biomarker for diagnosing acute LNB. It was a useful indicator in our investigation and might be a suitable indicator for diseases caused by spirochetes in general, as was already shown for *B. burgdorferi* and *Treponema pallidum* (*7*). Furthermore, the amounts of this chemokine might be used to assess the success of therapy because it declined rapidly in the patient we report after therapy began (Table). In contrast to the lack of sensitivity of culture and PCR for detecting *B. burgdorferi* in CSF, *B. miyamotoi*, as a member of the relapsing fever spirochete group, was detectable by microscopy and by PCR in the CSF in this patient, as well as in the 2 previously reported (*4**,**5*). Darkfield microscopy or staining with acridine orange might be required to increase sensitivity. In all 3 cases, PCR targeting the panbacterial 16S rRNA followed by sequencing showed the causative species. Our report of *B. miyamotoi* infection in a patient from Germany indicates that this emergent agent of central nervous system infection can be diagnosed by using existing methods if clinicians are aware of it.

Technical Appendix GenBank accession numbers for nucleotide sequence of *Borrelia miyamoto*i, Elsteraue, Germany, 2015.
