# Supplementary material for: Borrelia miyamotoi–Associated Neuroborreliosis in Immunocompromised Person
Source: Emerg Infect Dis. 2016 Sep;22(9):1617–20. doi: 10.3201/eid2209.152034 (PMC4994329; doi:10.3201/eid2209.152034)
Supplement: Technical Appendix — GenBank accession numbers for nucleotide sequence of Borrelia miyamotoi, Elsteraue, Germany, 2015. [file 15-2034-Techapp-s1.pdf]

Article DOI: http://dx.doi.org/10.3201/eid2208.152034

Borrelia miyamotoi–Associated Neuroborreliosis in Immunocompromised Persons

Technical Appendix

GenBank Accession Numbers for Nucleotide Sequence of *Borrelia miyamotoi*, Elsteraue, Germany, 2015

BankIt1864937	KT932822	16srRNA
BankIt1864947 Seq1	KT932823	flaB
BankIt1864947 Seq2	KT932824	clpA
BankIt1864947 Seq3	KT932825	clpX
BankIt1864947 Seq4	KT932826	pepX
BankIt1864947 Seq5	KT932827	pyrG
BankIt1864947 Seq6	KT932828	recG
BankIt1864947 Seq7	KT932829	rplB
